# Widespread activation of antisense transcription of the host genome during herpes simplex virus 1 infection

**DOI:** 10.1186/s13059-017-1329-5

**Published:** 2017-10-31

**Authors:** Emanuel Wyler, Jennifer Menegatti, Vedran Franke, Christine Kocks, Anastasiya Boltengagen, Thomas Hennig, Kathrin Theil, Andrzej Rutkowski, Carmelo Ferrai, Laura Baer, Lisa Kermas, Caroline Friedel, Nikolaus Rajewsky, Altuna Akalin, Lars Dölken, Friedrich Grässer, Markus Landthaler

**Affiliations:** 1 Berlin Institute for Medical Systems Biology, Max-Delbrück-Center for Molecular Medicine in the Helmholtz Association, Robert-Rössle-Strasse 10, 13125 Berlin, Germany; 2 Institute of Virology, Saarland University Medical School, Kirrbergerstrasse, Haus 47, 66421 Homburg/Saar, Germany; 3 Institut für Virologie und Immunbiologie, Julius-Maximilians-Universität Würzburg, Versbacherstr. 7, 97078 Würzburg, Germany; 4 Department of Medicine, University of Cambridge, Addenbrookes Hospital, Box 157, Hills Rd, Cambridge, CB2 0QQ UK; 5 Present address: AstraZeneca, Darwin Building, 310 Cambridge Science Park, Cambridge, CB4 0WG UK; 6 Institut für Informatik, Ludwig-Maximilians-Universität München, Amalienstraße 17, 80333 München, Germany; 7 IRI Life Sciences, Institute für Biologie, Humboldt Universität zu Berlin, Philippstraße 13, 10115 Berlin, Germany

**Keywords:** Herpes, Virus, Antisense, Transcription, lncRNA, ICP4, BBC3, NFKB

## Abstract

**Background:**

Herpesviruses can infect a wide range of animal species. Herpes simplex virus 1 (HSV-1) is one of the eight herpesviruses that can infect humans and is prevalent worldwide. Herpesviruses have evolved multiple ways to adapt the infected cells to their needs, but knowledge about these transcriptional and post-transcriptional modifications is sparse.

**Results:**

Here, we show that HSV-1 induces the expression of about 1000 antisense transcripts from the human host cell genome. A subset of these is also activated by the closely related varicella zoster virus. Antisense transcripts originate either at gene promoters or within the gene body, and they show different susceptibility to the inhibition of early and immediate early viral gene expression. Overexpression of the major viral transcription factor ICP4 is sufficient to turn on a subset of antisense transcripts. Histone marks around transcription start sites of HSV-1-induced and constitutively transcribed antisense transcripts are highly similar, indicating that the genetic loci are already poised to transcribe these novel RNAs. Furthermore, an antisense transcript overlapping with the BBC3 gene (also known as PUMA) transcriptionally silences this potent inducer of apoptosis *in cis*.

**Conclusions:**

We show for the first time that a virus induces widespread antisense transcription of the host cell genome. We provide evidence that HSV-1 uses this to downregulate a strong inducer of apoptosis. Our findings open new perspectives on global and specific alterations of host cell transcription by viruses.

**Electronic supplementary material:**

The online version of this article (doi:10.1186/s13059-017-1329-5) contains supplementary material, which is available to authorized users.

## Background

Herpesviruses are a family of enveloped DNA viruses which can infect a wide range of animal species. Herpes simplex virus type 1 (HSV-1) is a human pathogen, together with seven other viruses from the alpha, beta, and gammaherpesvirinae. HSV-1 is prevalent worldwide, with more than half of the population latently infected [1]. Beyond its own clinical importance, HSV-1 is used as a model for herpesviruses due to its easy application in the laboratory. A hallmark of HSV-1 is the “host cell shutoff” during lytic infection [2]. This widespread decrease of the expression of host cell genes is achieved at different stages of the RNA life cycle. First, transcription of host cell genes is impaired by reduced levels [3] and lower chromatin occupancy of RNA Pol II [4]. RNA splicing is then modulated by the viral protein ICP27 [5]. Finally, matured mRNAs are cleaved by the viral vhs/SOX endonuclease, and thus targeted for degradation [6, 7]. Recently, another RNA-related aberrance was observed in HSV-1-infected cells: about half of the transcribed host cell genes experience transcriptional read-through [8].

Transcription outside of annotated genes, sometimes called “pervasive transcription”, has attracted increasing interest in the past years [9, 10]. First, this term describes transcription from loci not immediately adjacent to known genes, giving rise to long non-coding RNAs (lncRNAs) or enhancer-derived RNAs (eRNAs). Second, antisense transcripts can originate from promoters of annotated genes [11, 12]. Third, transcripts can fully or partially overlap annotated genes, operating in a wide range of mechanisms, from chromatin remodeling to translation [13–16]. Here, we focus on the second and third categories, which we subsume for clarity of presentation as “antisense transcripts” in our text.

While antisense transcription from viral genomes has been described previously, e.g., in HIV-1 [17], and was also observed in our sequencing data from the HSV-1 genome, to our knowledge, modulation of host cell antisense transcription by virus infections has not been studied so far. Here, we set out to investigate antisense transcription of the host genome after HSV-1 infection, and identified around 1000 antisense transcripts specifically upregulated upon HSV-1 infection. Notably, most of these transcripts are not listed in existing genome annotations. Antisense transcription starts very early in infection. Using replication inhibitor and knockout viruses we show that overexpression of viral ICP4 alone is sufficient to induce expression of a subset of antisense transcripts. Analysis of published RNA-seq data from other herpesviruses revealed that, while induction of a subset of antisense transcripts is conserved within the *Alphaherpesvirinae* subfamily, infection with more distantly related herpesviruses does not lead to detectable upregulation of antisense transcripts.

Using a reporter assay, we showed that the sequence region upstream of the BBC3as antisense transcript functions as a promoter induced upon infection. Furthermore, we provide evidence that the induced antisense transcript impairs transcription of the BBC3 sense mRNA *in cis*. We propose that induction of antisense transcripts represents a previously undescribed strategy of how the virus modulates host gene expression.

## Results

### Detection of antisense transcripts in RNA-seq data from HSV-1-infected cells

**Fig. 1:**
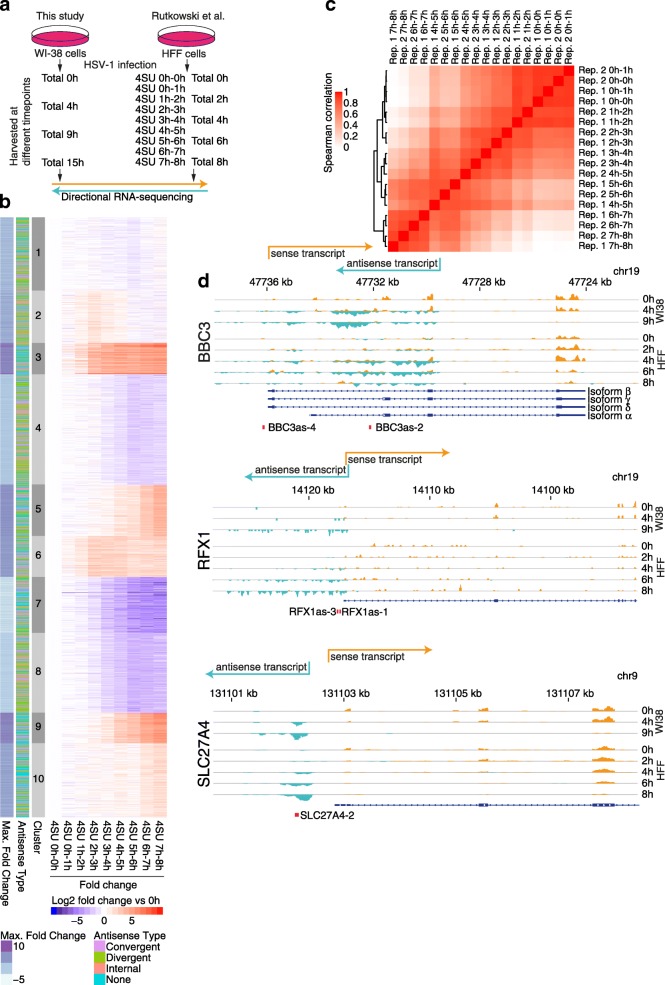
Characterization of antisense transcripts. **a** Overview of RNA-sequencing data used in this study. In addition to published data, we generated RNA-sequencing data from poly(A)-selected total RNA from HSV-1-infected WI-38 cells. **b** Clustering of antisense transcripts. Antisense transcripts were clustered based on the fold change between pulse labeling sequencing data at different timepoints after infection compared to mock-infected cells. **c** Clustering of correlations of antisense transcript expression values between timepoints and biological replicates. **d** Examples of antisense transcripts, from *top* to *bottom*: BBC3 antisense (internal), RFX1 antisense (convergent), divergent SLC27A4as. Coverage profiles for poly(A)-selected (WI-38 cells) and Ribozero treated (human foreskin fibroblast (HFF) cells) total RNA-sequencing data are shown. Sense genes are depicted in *orange* running *left* to *right*, antisense transcripts in *cyan* running *right* to *left*. Respective transcription starts and chromosome regions are indicated. Refseq annotations are shown in *dark blue*, locations of RT-qPCR primer pairs in *red*

To investigate transcriptional regulatory events in HSV-1-infected cells we sequenced mRNA from primary lung fibroblasts (WI-38 cells) at different timepoints until late into the lytic infection cycle (Fig. 1a). Two major phenomena emerged when analyzing host cell transcripts upon infection, namely transcriptional read-through as observed previously [8] and the widespread induction of antisense transcription.

For the detection of antisense transcripts, we additionally relied on directional RNA-sequencing data from human foreskin fibroblasts of rRNA-depleted total RNA and 4-thiourdine pulse-labeled and subsequently purified newly transcribed RNA (4sU-RNA) [8] (Fig. 1a). For maximal sensitivity, the data from 4sU-RNA was analyzed with a newly developed algorithm to detect antisense transcripts. After initial definition of transcribed regions outside of annotated genes, transcriptional read-throughs were filtered out (see “Methods”). Using a running sum algorithm, we defined 12,863 transcribed regions either annotated as antisense transcripts in Ensembl or not annotated at all. Of these, 9765 regions were designated as read-through regions. The remaining 3098 loci were grouped by their expression profiles into ten clusters (Fig. 1b; Additional file 1: Figure S1a; Additional file 2: Table S1). Based on their location, transcripts were categorized as divergent, convergent, or internal antisense transcripts (Fig. 1d), or, if the transcription start site (TSS) of the antisense transcript was not within 10 kb of a TSS of a transcript on the opposite strand, and if it did not overlap a gene body, it was categorized as “undefined”.

Of the 3098 antisense transcripts, 1517 showed at least two-fold upregulation upon infection. Comparing them with a recently published, comprehensive atlas of human non-coding RNAs [18], we found that only 384 of these begin within less than 100 bp from an annotated TSS, indicating that most of the upregulated transcripts were previously undescribed. As 503 of the 1517 upregulated transcripts were categorized as “undefined”, this leaves around 1014 antisense transcripts upregulated by the HSV-1 infection.

To determine when during infection the antisense transcripts emerge, we clustered the correlation coefficients of their expression values at the different timepoints (Fig. 1c). As expected, biological replicates were highly correlated and the clustering matched the time course of infection. Importantly, sequencing data obtained from RNA labeled 1 to 2 h after infection was separated from the mock and 0–1-h timepoint, indicating that antisense transcripts are already expressed very early in infection.

We selected 12 antisense transcripts for further analyses, focusing on upregulated antisense transcripts from clusters 3 and 6 (Table 1), including the SNX6 antisense transcript, defined by the algorithm as a read-through transcript from a U1 snRNA gene (Additional file 1: Figure S1a). Sequence coverage profiles of the antisense transcripts belonging to different categories are shown in Fig. 1d and Additional file 1: Figure S1a. The maximal RPKM values for the antisense transcripts in relation to RPKM values of the cellular genes in uninfected cells are shown in Additional file 1: Figure S1b.

**Table 1:** Transcripts selected for detailed analysis

Transcript	Type	Cluster
BBC3as	Internal	3
C1orf159as	Convergent, spliced 5′ UTR, asTSS in 5′ UTR intron	3
EFNB1as	Divergent	3
FOXO3as	Convergent, spliced 5′ UTR, asTSS in 5′ UTR intron	3
IER2as	Convergent, spliced 5′ UTR, asTSS in 5′ UTR intron	6
IFFO2as	Overlap or divergent from ALDH4A1	3
ING1as	Convergent, spliced 5′ UTR, asTSS in 5′ UTR intron	3
MEGF6as	Internal	6
NFKB2as	Convergent, spliced 5′ UTR, asTSS in 5′ UTR intron	3
RFX1as	Convergent, spliced 5′ UTR, asTSS in 5′ UTR intron	3
SLC27A4as	Divergent	3
SNX6as	(Read-through transcript)	NA

*asTSS* antisense transcription start site, *NA* not applicable

Generally, we observed that the antisense transcripts were not spliced. Noteworthy, antisense transcripts were found in the poly(A)-selected RNA, suggesting that they are polyadenylated.

The identified antisense transcripts can overall be classified into: a) divergent antisense transcripts, where the sense and antisense transcripts likely start from the same promoter region, but do not overlap; b) convergent antisense transcripts, where the 5′ ends of the antisense and the canonical sense transcript overlap; and c) internal antisense transcripts, where several exons of the canonical sense transcript are overlapping with the antisense transcript.

Taken together, we detected 3098 novel antisense transcripts in strand-specific RNA sequencing data, thereby expanding the catalog of lncRNAs [19]. Of these antisense transcripts, 1014 showed increased expression upon HSV-1 infection.

### Validation and expression dynamics of antisense transcripts

**Fig. 2:**
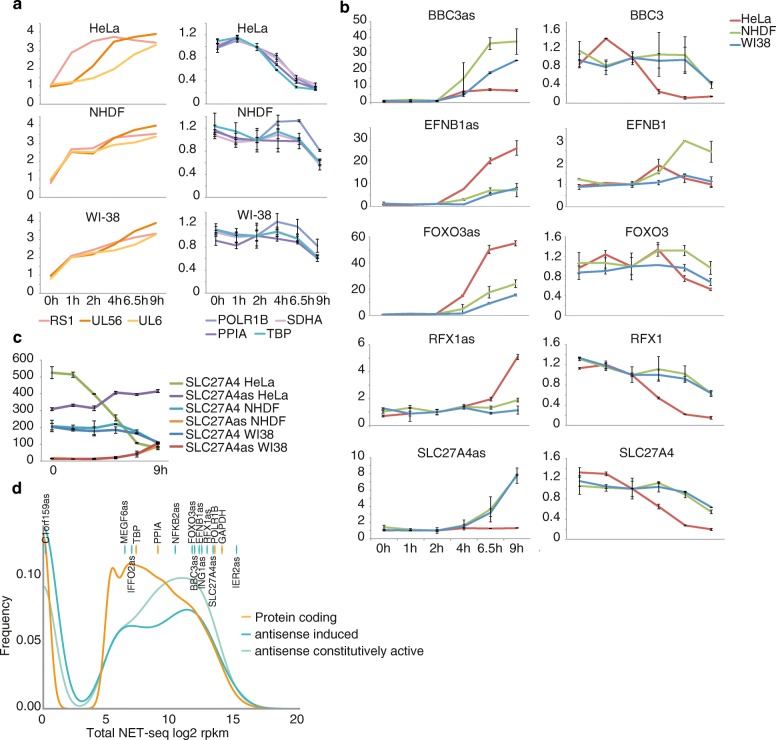
Validation of antisense transcripts. **a** Nanostring nCounter assays: controls. RNA was isolated from the three indicated cell lines at different timepoints post-infection and used for Nanostring nCounter assays. Experiments were performed with one measurement each from two biological replicates and scaled to the 2 h post-infection (hpi) timepoint after normalization using the provided control spike-ins. Values for HSV-1 mRNAs are shown as log(10) transformed normalized Nanostring counts. *Error bars* represent standard deviations. **b** Nanostring nCounter assays: antisense transcripts and corresponding sense mRNAs. Assays were performed as in **a**. **c** Absolute counts of SLC27A4 antisense and sense. To compare the three cell lines, absolute counts after technical normalization using the provided control spike-ins are shown. *Error bars* represent standard deviations. **d** Expression of antisense transcripts in total NET-seq data. Shown is the distribution of antisense transcripts and protein coding genes as lines, with the values of the 12 selected antisense transcripts and housekeeping genes marked on top

RNA sequencing data suggested that antisense transcription started shortly after infection. Therefore, we focused on early timepoints of infection (Table 1). To validate and quantify antisense transcription, we performed gene expression measurements using Nanostring nCounter assays, which are inherently strand-specific and thus highly suitable to probe antisense transcripts. We measured the expression of the 12 antisense transcripts listed in Table 1 in three different human cell lines (HeLa, WI-38, and NHDF) infected with HSV-1 (Fig. 2a; Additional file 1: Figure S2b). These analyses provided further confirmation of antisense transcription, and a comparison of the relationship between the progress of infection in the various cell lines and the expression dynamics of the antisense transcripts. First, we compared the mRNA expression changes of transcript-encoding housekeeping genes and HSV-1 mRNAs between the three cell lines (Fig. 2a). Values for HSV-1 mRNAs are shown as log(10) transformed normalized Nanostring counts. As expected, we observed that the progression of HSV-1 mRNA counts was similar in the two primary fibroblast cell lines NHDF and WI-38 (Fig. 2a, two bottom left panels), while HeLa cells (Fig. 2a, top left panel) displayed a different profile. Viral host cell shutoff [2], as measured by the abundance of the housekeeping genes, became measurable in HeLa cells already 4 h after infection (Fig. 2a, top right panel). In the two fibroblast cell lines, however, levels of the measured mRNAs did not change until 6.5 h after infection (Fig. 2a, two bottom right panels).

Confirming the RNA sequencing results, Nanostring counts for antisense transcripts increased during infection (Fig. 2b; Additional file 1: Figure S2c). However, some remarkable differences between cell lines and individual transcripts were observed. The BBC3as transcript (Fig. 2b, top panel) already started to accumulate 1 h after infection in HeLa cells, yet it eventually reached higher levels in the two fibroblast cell lines. Generally, a delayed induction of the antisense transcripts could be monitored in these cells, reflecting the slower progression of infection, which is also observed for the housekeeping genes (Fig. 2a, right). RFX1as and SLC27Aas are the only antisense transcripts of the 12 tested where strong cell line specificities could be observed. RFX1as was only induced in HeLa cells (Fig. 2b, fourth panel), while SLC27A4as was already present in untreated HeLa cells, as shown by comparison of normalized Nanostring counts in the different cell lines (Fig. 2c). Still, there was a relative increase in expression upon infection, although it was considerably lower compared to WI-38 and NHDF. Interestingly, the beginning of the SLC27A4 antisense transcript partially overlapped in some annotations with a tRNA gene. While the coverage profile did not suggest that the antisense transcript is a tRNA, there was no apparent transcription termination at the RNA Pol III terminator. This observation could indicate that the tRNA and the antisense transcript share a common promoter region.

We further validated the antisense transcripts using RT-qPCR from uninfected (0 h) and infected HeLa cells (Additional file 1: Figure S2a). Many of the tested transcripts could not be reliably detected in uninfected cells. Therefore, we normalized qRT-PCR data to a *Drosophila melanogaster* total RNA spike-in and expression levels at the 2-h timepoint. We included two host MAP kinase target genes as well as pre-miR-183, which are induced during lytic HSV-1 infection [20, 21].

We also considered whether the observed antisense transcripts are a consequence of the reported widespread transcriptional read-through in HSV-1-infected cells [8]. First, we compared the read-through transcripts with the antisense transcripts in the 4sU-seq data (Additional file 1: Figure S2c). There, the two transcript classes showed different expression dynamics. Furthermore, two read-through transcripts were measured using RT-qPCR from the same samples used in Additional file 1: Figure S2a (Additional file 1: Figure S2d). We observed that for these two targets, the qPCR amplicons could only be reproducibly detected at 4 hpi (data not shown), with a strong signal increase from 4 to 6 hpi. Again, this dynamic behavior is different from all antisense transcripts, which also suggests that the antisense transcripts under scrutiny here are not products of the transcriptional read-through observed in HSV-1-infected cells.

To address the question of whether the induced antisense transcripts might also be transcribed in uninfected cells, we used NET-seq data [22], which is currently one of the most sensitive methods for detection of transcription. RPKM values were calculated from the HeLa total NET-seq control samples, and log2 transformed numbers are plotted in Fig. 2d. A threshold of 3 was applied to separate noise values. Out of 1014 induced antisense transcripts, 762 (75%) have a log2 RPKM in NET-seq larger than this threshold. The majority of induced antisense transcripts are therefore already transcribed in uninfected cells at levels comparable to protein coding genes, but are not detectable in high-throughput sequencing of steady state or newly synthesized RNA (4sU-seq).

Finally, we investigated whether the antisense transcripts could be stabilized upon HSV-1 infection. To this end, we analyzed nucleoplasmic RNA in HeLa cells upon depletion of the exosome component EXOSC3 (also known as RRP40) [22]. Indeed, we could observe at least twofold upregulation of 629 of the 1517 transcripts upregulated in HSV-1-infected cells, among them, e.g., BBC3as (Additional file 1: Figure S2e). However, our antisense detection algorithm identified 12,417 antisense transcripts upregulated in EXOSC3-depleted cells compared to control cells, among them the recently described promoter upstream transcripts [23, 24]. This discrepancy indicates that reduced exosome activity is unlikely a cause of the induction of antisense transcription by HSV-1 infection.

Taken together, we validated the presence of antisense transcripts using two low-throughput methods in three different cell lines. Notably, SLC27A4as was also detected in uninfected HeLa but not NHDF or WI-38 cells. Furthermore, antisense transcripts show expression dynamics distinct from poly(A) read-through and thus represent an independent entity of transcripts.

### Viral factors induce antisense transcripts by different mechanisms

**Fig. 3:**
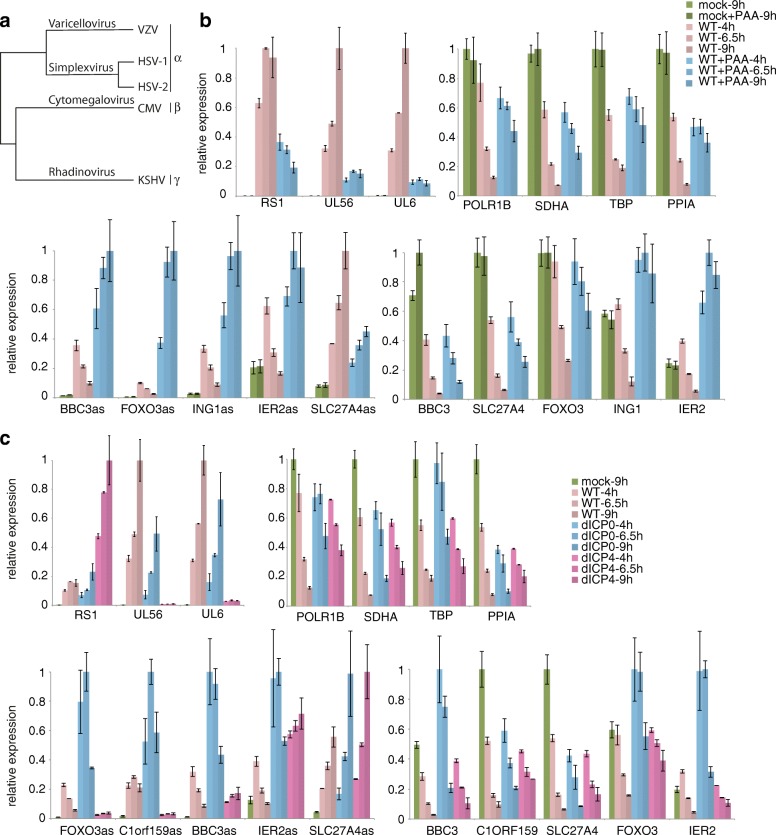
Antisense expression using phosphonoacetic acid (PAA) and knockout viruses. **a** Simplified phylogenetic tree showing the analyzed herpesviruses. **b** Nanostring nCounter profiling with the replication inhibitor PAA. RNA was collected from HFF cells at different timepoints post-infection with or without the HSV-1 replication inhibitor PAA. Infection and host cell shutoff were tracked using selected HSV-1 transcripts and housekeeping genes (*top panels*). A subset of antisense transcripts and corresponding host genes (all 12 antisense transcripts in Additional file 1: Figure S3) are shown in the *two lower panels*. Experiments were performed with one measurement each from two biological replicates and scaled to the 2 hpi timepoint after normalization using the provided control spike-ins. *Error bars* represent standard deviations. All values are scaled to the largest value for the same transcripts. Antisense and sense transcripts are sorted by expression profile. **c** Nanostring nCounter profiling using ICP0 and ICP4 knockout viruses. RNA was collected from HFF cells at different timepoints post-infection with wild-type (*WT*), ∆ICP0, or ∆ICP4 virus. Infection and host cell shutoff were tracked using selected HSV-1 transcripts and housekeeping genes (*top panels*). A subset of antisense transcripts and corresponding host genes (all 12 antisense transcripts in Additional file 1: Figure S3) are shown in the two lower panels. Experiments were performed with one measurement each from two biological replicates and scaled to the 2 hpi timepoint after normalization using the provided control spike-ins. *Error bars* represent standard deviations. All values are scaled to the largest value for the same transcripts. Antisense and sense transcripts are sorted by expression profile

To investigate the mechanisms and factors that induce antisense transcript transcription, we analyzed RNA-seq data from infections with other herpes viruses, performed a series of HSV-1 infections of human foreskin fibroblast (HFF) cells under modifying conditions (Fig. 3), and quantified sense and antisense transcript expression using Nanostring nCounter assays. Humans can be infected by eight different herpesviruses (Fig. 3a), with HSV-1 belonging to the *Alphaherpesvirinae* subfamily. We therefore investigated whether infections by other human herpesviruses would also induce antisense transcripts.

To that end, we used published RNA-seq data for the alphaherpesvirus varicella zoster virus (VZV) [25] as well as the betaherpesvirus human cytomegalovirus (HCMV) [26], and the gammaherpesvirus Kaposi’s sarcoma-associated herpesvirus (KSHV) [27]. Due to differences in sequencing depths, and since the VZV sequencing is unstranded, a fully quantitative comparison was not possible. For KSHV-infected cells we did not find any indication for the antisense transcripts induced by HSV-1. In VZV-infected differentiated keratinocytes, however, we observed induction of several hundred antisense transcripts. In HCMV-infected cells only a few antisense transcripts were detected, with the important exception of SLC27A4as, which was shown to be ICP4-independent (Fig. 3c). Within the *Alphaherpesvirinae* family, the *Herpes simplex* genus includes a second member that infects humans, namely HSV-2. We therefore probed RNA from HSV-2-infected NHDF cells with our Nanostring assay and observed a similar pattern of antisense transcripts compared to infection with HSV-1 (Additional file 1: Figure S3c).

In order to test how the expression of the antisense transcripts depended on the progression of lytic infection, we used the HSV-1 DNA replicase inhibitor phosphonoacetic acid (PAA), which prevents expression of viral late genes. Consequently, levels of viral transcripts were much lower (Fig. 3b, first panel). Due to the attenuated infection, HSV-1 induced host shutoff, as measured by RNA levels of the housekeeping genes POLR1B, SDHA, TBP, and PPIA, which were reduced. While the values with and without PAA at 4 hpi were comparable, at 6.5 and 9 hpi these transcripts were about twofold to threefold higher, respectively (Fig. 3b, second panel). Interestingly, most, but not all, antisense transcripts were more abundant with PAA (Fig. 3b, third panel; Additional file 1: Figure S3a). Some antisense transcripts, such as BBC3as, FOXO3as, IER2as, and ING1as, were several times more abundant in PAA-treated cells (Fig. 3a), while SLC27A4as showed the opposite behavior, with higher levels in the absence of PAA.

The corresponding sense transcripts showed diverse response patterns (Fig. 3b, fourth panel; Additional file 1: Figure S3a). IER2 is induced by the MAP kinase pathway [28], which is in turn activated by the infection itself [29]. Similarly to IER2, the pro-apoptotic gene ING1 [30] was induced by MAP kinases as well, and, like IER2, showed higher mRNA levels at all timepoints after infection compared to uninfected cells under PAA treatment. This result indicates an induction of these genes by the virus infection, which is, however, counteracted by the host cell shutoff.

In addition, we measured the expression profiles in cells infected with ICP0 or ICP4 deletion viruses. ICP0 functions as a ubiquitin ligase and contributes to an active viral chromatin structure [31]. ICP4 is mainly considered as a regulator of transcription of the viral genome, interacting with several host cell transcription factors [32, 33]. The ICP0 knockout virus (deletion of the full gene) leads to an attenuated infection, i.e., slower progression and viral replication, but otherwise all viral genes are expressed and functional virions are produced. Without functional ICP4, however, the virus does not progress past expression of immediate early genes (the ICP4 deletion virus contains a short deletion in the N-terminus of the RS1 gene, leading to a frameshift and non-functional protein). In our assay, the late genes UL6 and UL56 consequently accumulated more slowly in the ICP0 deletion virus compared to the wild-type virus, and were absent in the ICP4 deletion virus. ICP4/RS1, itself an immediate early gene, was constantly induced and accumulated (Fig. 3c, first panel). The host cell shutoff proceeded in a comparable manner between the two deletion strains, but slower than in the wild-type virus (Fig. 3c, second panel).

The antisense transcripts again showed different profiles (Fig. 3c, third panel; Additional file 1: Figure S3b). BBC3as, C1orf159as, and FOXO3as were more abundant upon infection with the ICP0 deletion virus compared to the wild-type virus; the difference was similar to that with and without PAA (Additional file 1: Figure S3a, b). Again, IER2as and SLC27A4as transcripts showed distinct expression profiles. For both antisense transcripts, induction was stronger in the ICP4 knockout virus compared to the wild-type virus, with the signals from the two knockout viruses being similar to each other. Because only viral immediate early genes are expressed when using the ICP4 knockout virus, and IER2as and SLC27A4as were detectable under this condition, this difference indicates that IER2as and SLC27A4as are induced by immediate early genes, or by a cellular mechanism triggered by the infection. On the other hand, transcription of BBC3as, C1orf159as, and FOXO3as is likely turned on by early genes or by ICP4 itself.

### Overexpression of ICP4 is sufficient for induction of some antisense transcripts

**Fig. 4:**
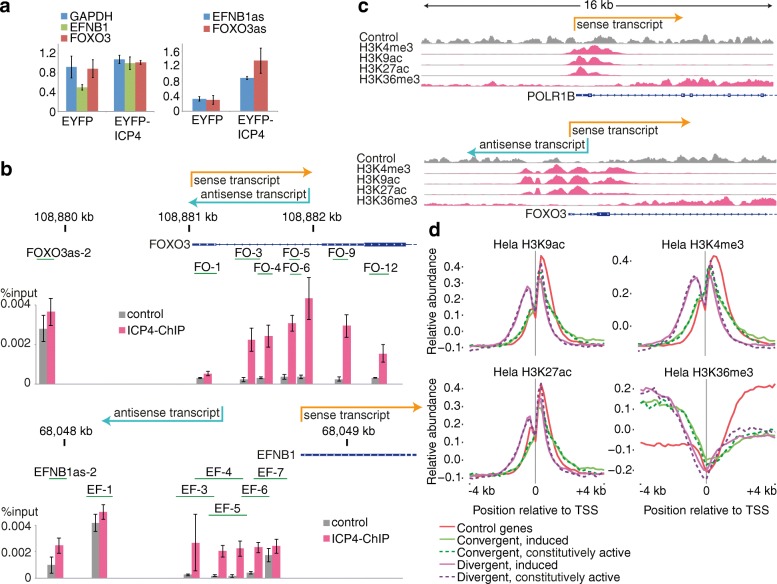
Antisense transcript promoters are already poised for transcription. **a** Antisense transcript induction upon ICP4 overexpression. EYFP-ICP4 or the EYFP-only control plasmid were transfected into HeLa cells and RNA was isolated and subjected to random hexamer-directed RT followed by qPCR with primer pairs as indicated. Experiments were performed with two measurements each from two biological replicates and normalized to the wild-type EYFP-ICP4 value using the *D. melanogaster* spike-in RNA. Error bars represent standard deviations. In control cells, the FOXO3 antisense transcript could not be reliably quantified since the amplicon was not present in all samples (Additional file 1: Figure S4). **b** ChIP-qPCR with ICP4-3xflag. ChIP was performed from ICP4-3xflag-transfected HeLa cells, and untransfected cells as control, followed by qPCR using different amplicons, indicated as *green lines*, around the transcription start sites of FOXO3 and EFNB1. Values are presented as percentage input, and averaged from two measurements each from two biological replicates. Error bars represent standard deviations. **c** Histone marks at promoter regions. Encode Broad histone marks are shown around the transcription start sites of the unidirectional POLR1B and the bidirectional FOXO3 genes. Transcripts of the sense transcripts and the inducible FOXO3 antisense transcript are indicated. **d** Histone mark metaplots. Metaplots were generated for four histone marks around the transcription start sites of inducible and constitutively transcribed antisense transcripts, together with 1000 unidirectional control genes

Because some antisense transcripts were not detectable in cells infected with the ∆ICP4 virus (Fig. 3c), we investigated whether ICP4 itself is sufficient to induce some antisense transcripts. To this end, we overexpressed EYFP-tagged ICP4 in HeLa cells, using EYFP only overexpression as a control (Fig. 4a; Additional file 1: Figure S4). The RNA isolated from these cells was probed using Nanostring nCounter assays (Additional file 1: Figure S4b) and directional high-throughput RNA-sequencing (Additional file 2: Table S1). For two antisense transcripts, EFNB1as and FOXO3as, we also performed RT-qPCR (Fig. 4a, right panel). The FOXO3 antisense transcript was not reliably detectable in EYFP-transfected cells, based on agarose gel electrophoresis of the qPCR products (Additional file 1: Figure S4a). Interestingly, while the FOXO3 sense mRNA remains unaltered upon EYFP-ICP4 infection, levels for the EFNB1 mRNA showed a twofold increase. This indicates that ICP4 might increase transcription in both directions for EFNB1/EFNB1as, but not for FOXO3/FOXO3as.

To further investigate whether ICP4 directly binds to the promoter region of target genes, we performed ICP4 ChIP-qPCR, as previously performed for RCL1 [34]. The strongest enrichment for the ICP4-ChIP was indeed found in both cases at the beginning of the antisense transcript (Fig. 4b).

### Histone marks are similar for induced and constitutively active antisense transcripts

Antisense transcripts appear very early in infection and can be found widespread throughout the host cell genome. To address the question of whether the virus starts transcription from primed regions, we went on to examine ENCODE histone marks [35] around TSSs. Figure 4c depicts HeLa histone marks of active promoters (H3K4me3, H3K9ac, H3K27ac) and actively transcribed gene bodies (H3K36me3) of a unidirectional gene, POLR1B, and FOXO3, which bears a HSV-1 inducible divergent antisense transcript. While the POLR1B gene showed the expected profile for a unidirectional gene, the island of activating promoter histone marks was much larger around the FOXO3 TSS, reminiscent of bidirectional promoters [36]. We extended this analysis to include all detected antisense transcripts (Fig. 4d; Additional file 1: Figure S4C). In total, five sets of genes were examined. First, 1000 unidirectional control genes were analyzed. For the convergent and divergent antisense transcripts detected here, we subsequently analyzed histone marks around the TSS of induced and uninduced antisense transcripts separately.

Control genes showed a strong single peak at the TSSs for the three active promoter marks, and a strong increase of H3K36me3 occupancy for active gene bodies downstream of the TSS. Genes having divergent antisense transcripts showed the expected bimodal distribution for active promoters, but, remarkably, the induced and constitutive transcripts did not show any difference. This observation was true for H3K36me3 as well, which showed high levels in both directions from the TSS.

Genes with convergent transcripts also showed a single peak for the active promoter marks, but with a shoulder downstream of the TSS, which could reflect the transcription start for the antisense transcript. Again, inducible and constitutive transcripts showed the same profiles. These results indicate that active promoter marks enabling antisense transcription are already present in the uninfected cells.

### BBC3as is transcribed from a HSV-1-dependent promoter and inhibits BBC3 sense *in cis*

To investigate the physiological role of antisense transcripts, we focused on BBC3as, which was among the most highly expressed, and dynamically induced antisense transcripts (Additional file 1: Figure S1b). BBC3as is antisense to the strong apoptosis inducer BBC3, also known as PUMA [37]. Concomitantly, viruses try to counter cell death in different ways to enable maximal replication [38].

**Fig. 5:**
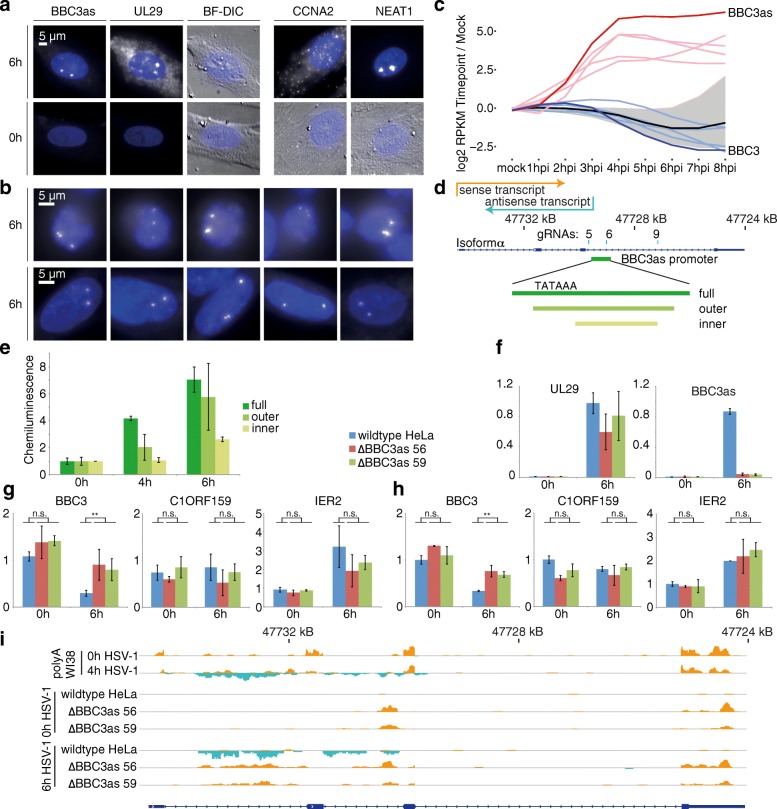
Evidence for inhibition of sense transcription by BBC3as. **a** Single-molecule FISH. HSV-1-infected cells (6 h) and non-infected controls (0 h). As a reference, non-infected controls were stained for an example mRNA (CCNA2) and lncRNA (NEAT1). Note that because of lower (mRNA) or higher (lncRNA) expression, exposure times and image settings had to be adjusted and are not directly comparable between panels **a** and **b**. **b** Comparison of BBC3as in HeLa and NHDF cells. Nuclear foci in infected (6 h) HeLa (*upper row*) and NHDF (*lower row*) cells. **c** Profiling sense and antisense transcripts in pulse sequencing data. Shown are log2 fold changes compared to mock-treated cells at different timepoints. *Black line*, median of all genes; *gray area*, 25/75% quantiles; *unlabeled light blue*/*light red* represent the other four sense/antisense transcripts from **a**. **d** BBC3 locus. Transcription start of sense and antisense RNA and guide RNAs used for CRISPR cell lines are indicated. The putative BBC3as promoter region and subregions used for the promoter assay in **d** are shown as *green bars*. Note that only the full and outer constructs contain the TATAAA sequence. **e** Reporter assay of the putative BBC3as promoter. The sequences indicated in **c** were cloned in front of the Firefly luciferase, with the Renilla luciferase on the same plasmid for normalization. Shown are chemiluminescence Renilla/Firefly ratios from two biological replicates scaled to the 0-h timepoint. *Error bars* represent standard deviations. **f** RT-qPCR on total RNA. RNA was isolated from HeLa cells labeled for 20 minutes with 4sU. Shown is total RNA from uninfected cells and 6 h post-infection, subjected to random hexamer-directed RT followed by qPCR with primer pairs as indicated. Experiments were performed with two measurements each from two biological replicates and normalized to the wild-type HeLa 0-h value using the *D. melanogaster* spike-in RNA. *Error bars* represent standard deviations. **g** RT-qPCR on newly synthesized RNA. Labeled, i.e., newly synthesized RNA, was isolated and subjected to RT-qPCR as in **f**. Significance levels were calculated using a *t*-test with equal variance levels. **p ≤ 0.01; not significant (*n.s*.), *p* > 0.05. **h** Nanostring nCounter assays on newly synthesized RNA. Experiments were performed with one measurement each from two biological replicates and scaled to the wild-type HeLa 0-h value after normalization using housekeeping genes. *Error bars* represent standard deviations. Significance levels were calculated using a *t*-test with equal variance levels. **p ≤ 0.05; not significant (*n.s*.) *p* > 0.05. **i** RNA-sequencing coverage profiles of the BBC3 locus of wild-type HeLa cells and the two BBC3as promoter knockout cell lines

As a first step, we localized the BBC3 antisense transcript using single molecule FISH [39, 40]. We used diploid NHDF cells to probe for BBC3as, simultaneously detecting the HSV-1 UL29 transcript as a positive control for the infection (Fig. 5a). The signal for BBC3as was only detected in UL29-positive infected cells, indicating probe specificity. UL29 RNA was present at high levels in the cytoplasm, with some strong foci in the nucleus likely representing sites of viral transcription. BBC3as showed two such nuclear foci, but at different positions. As controls, we used CCNA2 mRNA, which localizes to the cytoplasm, as well as the lncRNA NEAT1 (Fig. 5a), which was previously shown to also localize in nuclear foci [40].

While the signal from the CCNA2 probes likely represented monodispersed single RNA molecules, the bright nuclear foci for NEAT1 and BBC3as likely indicate the accumulation of multiple RNA molecules [40]. Because HeLa cells are triploid for the BBC3 locus [41], three foci were visible in HeLa cells compared to two in NHDF cells, suggesting BBC3as accumulated at the site of transcription (Fig. 5b). In summary, single molecule RNA FISH data indicated that the BBC3as transcript accumulates at discrete foci in HSV-1-infected cells, likely at the locus which encodes the pro-apoptotic gene BBC3 [37]. In such a scenario, BBC3as might inhibit BBC3 at the level of transcription. Ectopic overexpression of the first 2191 nucleotides of the BBC3as led to a strong signal in multiple foci in the cytoplasm (Additional file 1: Figure S5a), a behavior that was also previously observed for lincRNA-p21 [42].

Using Nanostring quantification, BBC3 mRNA exhibited a stronger decrease in expression than mRNA derived from housekeeping genes, both with and without PAA treatment (Fig. 3b). Because BBC3as is induced early in infection (Additional file 1: Figure S2a; Fig. 3b), we reasoned that BBC3 is subject to active downregulation during the infection. For closer examination of BBC3 mRNA transcription, we considered the 4sU-seq data sampled at different timepoints after infection. Comparing the sense and antisense transcripts of BBC3 and the four other genes displayed in Fig. 3b, we observed that, after an initial increase at early timepoints, the expression for BBC3 was lower than for housekeeping genes (Fig. 5c). This observation again indicates that an active mechanism could impair BBC3 mRNA transcription, with the antisense transcript as a prime candidate to mediate a possible transcriptional interference.

To define the promoter region of the BBC3 antisense transcript, we performed dual luciferase promoter reporter assays using a region upstream of the BBC3as transcript, which contains two TATA boxes. Different genomic fragments from that region (Fig. 5d) were inserted upstream of an otherwise promoter-less luciferase gene. Luciferase activity was measured in uninfected and HSV-1-infected HeLa cells, showing a luciferase signal increase upon infection (Fig. 5e). This result confirms the existence of a promoter that is activated by the infection and would lead to the transcription of the BBC3as transcript.

Because our attempts to deplete the antisense transcript using siRNAs or gapmers were unsuccessful (data not shown), we went on to deplete BBC3as by excising the promoter region using Cas9 in combination with two different guide RNA pairs (5/6 and 5/9; Fig. 5d) from HeLa cells. As expected, BBC3as was largely undetectable in the two independently generated BBC3as promoter knockout cell lines (termed ∆BBC3as 56 and ∆BBC3as 59), as measured by RT-qPCR from total RNA (Fig. 5f). By contrast, viral UL29 mRNA levels were largely comparable between the HeLa and knockout cell lines. We then measured the expression levels of different transcripts in the newly transcribed RNA population using 4sU-tagging [43, 44]. C1ORF159 and IER2 levels change comparably upon infection in wild-type HeLa and BBC3as knockout cells, as measured by RT-qPCR (Fig. 5g) and Nanostring nCounter assays (Fig. 5h). However, the BBC3 mRNA synthesis showed a marked decrease upon infection in wild type, but not in BBC3as knockout cells. To further investigate the effect of the BBC3 antisense transcript on its sense counterpart, we performed directional high-throughput sequencing using RNA from wild-type and BBC3as knockout cells at 0, 2, 4, and 6 h after infection in two biological replicates. In Fig. 5i, one replicate for all three cell lines at 0 and 6 h after infection is shown. Only in the BBC3as knockout cells was there a remarkable increase of sequencing coverage in the sense direction in the first, slowly processed [45] half of the BBC3 gene upon infection. This result suggests that, in infected cells, BBC3 pre-mRNA is increasingly transcribed, but only in the absence of antisense transcription.

Taken together, we provide evidence that the BBC3as transcript is expressed upon HSV-1 infection and inhibits transcription of BBC3 *in cis*, and therefore enables the virus to suppress this potent inducer of apoptosis.

### NF-kappaB transcription factor genes show age-dependent induction of antisense transcripts in lipopolysaccharide-treated monocytes

The detection of thousands of upregulated antisense transcripts raises the question of whether these RNA transcripts can also be detected in other biological systems. If so, the HSV-1-induced antisense transcripts could be used as a template to study inducible antisense transcription elsewhere. We performed a literature search for the 12 antisense transcripts listed in Table 1 and found one of them, NFKB2as, to be induced by lipopolysaccharide (LPS) in macrophages [46]. We further investigated the presence of this antisense transcript in published RNA-sequencing data after LPS induction in monocytes, the precursor cells of macrophages [47]. Indeed, in monocytes from younger adults and older adults both NFKB2 sense and NFKB2 antisense were induced upon LPS treatment (Table 2; Additional file 1: Figure S1).

**Table 2:** NF-kappaB antisense transcripts after LPS treatment and HSV-1 infection

Gene	LPS antisense upregulated	LPS sense upregulated	HSV-1 antisense upregulated	HSV-1 sense upregulated
NFKB1	Adult and older adults yes, newborn no	Yes	Yes	No
NFKB2	Adult and older adults yes, newborn no	Yes	Yes	No
REL	Older adults weakly, adults and newborn no	Yes	No	No
RELA	No	Yes	Yes	No
RELB	No	Yes	Yes	No

In monocytes from newborns, only the sense NFKB2 is induced, to an extent comparable to adults and older adults, but not NFKB2as. The NFKB1/p105 antisense transcript shares the same properties. Interestingly, while in LPS-stimulated monocytes the respective host genes were upregulated together with the antisense transcripts, in HSV-1 infections only the antisense transcripts were upregulated. Among the three other NF-KB factors, REL has an annotated antisense transcript (official gene symbol LINC01185) that was weakly induced in monocytes from older adults, but not in any of the other samples examined here. RELA and RELB finally showed antisense transcripts induced in HSV-1-infected cells, but not in any of the monocyte samples. Again, all three Rel genes were induced in LPS-treated monocytes, but not in HSV-1-infected cells.

In summary, we show age-related antisense transcript induction for genes of the NF-kappaB transcription factor family in LPS-treated monocytes.

## Discussion

An initial analysis of RNA-seq data from HSV-1-infected human cells brought to our attention that, upon infection, antisense transcripts emerge at several loci in the host cell genome. An in-depth analysis of our own and published sequencing data showed indeed that this induction of antisense transcripts occurs throughout the genome, and 1517 such RNA species, most of them novel and not previously annotated, are induced or upregulated upon virus infection.

Validation of the discovered antisense transcripts using RT-qPCR and Nanostring nCounter assays showed different expression dynamics of antisense transcripts and their respective sense transcripts. However, we did not find significant correlations between sense and antisense transcription, indicating that, in general, their mechanisms for transcription initiation are independent of each other.

We investigated two possibilities concerning whether the observed antisense transcripts arise due to the perturbation of transcription-related processes. First, the antisense transcripts could emerge following stabilization of unstable transcripts. Second, they could show up due to impaired transcription termination [12], which could render short unstable transcripts longer and, concomitantly, also more stable.

Because we relied on sequencing data of pulse-labeled RNA for the bioinformatic detection of antisense transcripts, short-lived transcripts were preferentially detected and therefore could also be observed in uninfected cells.

To address the question of whether the antisense transcripts are in some way a consequence of the infection-induced transcriptional read-through, we looked at the expression dynamics of read-through transcripts and found that they emerge later in infection, but then with a very steep increase (Additional file 1: Figure S2c, d). Nevertheless, antisense transcripts that come up later in infection (e.g., cluster 5 in Fig. 1b) might still be a consequence of transcriptional read-through. We are currently dissecting the causes for this observation, which might provide a more comprehensive answer to the relationship between the two transcriptional phenomena observed in HSV-1-infected cells. In a broader context, the observation of antisense transcription and transcriptional read-through in HSV-1-infected cells could also help to elucidate the mechanisms underlying promoter directionality and transcription termination [12]. Importantly, some of the antisense transcripts appear to be upregulated in nucleoplasmic RNA from EXOSC3-depleted cells [22]. However, many antisense transcripts that are upregulated by exosome depletion become expressed in HSV-1-infected cells. A reduction of exosome components upon infection was not observed, on either the RNA [8] protein level [48], indicating a more complex relationship between exosome activity and HSV-1 infection.

The clustering of the expression dynamics of the antisense transcripts (Fig. 1b) already suggested that certain subsets were induced by different mechanisms. We therefore applied modified infection conditions, namely the replication inhibitor PAA and two knockout viruses (Fig. 3). This accentuated differences in expression dynamics of the antisense transcripts, like for SLC27A4as or IER2as. To further explore the different mechanisms causing antisense transcription, we first examined sequencing data from VZV, which belongs to the genus *Varicellovirus* within the alphaherpesviruses family, HCMV (beta), and KSHV (gamma). While we did not detect HSV-1-induced antisense transcripts in KSHV-infected cells, a subset of antisense transcripts was induced in VZV-infected cells. The SLC27A4as transcript, apparently independent of the viral protein ICP4 (Fig. 3), was one of the few induced also in CMV-infected cells. This is consistent as ICP4 is only present in alphaherpesviruses. Ectopic expression of ICP4 showed that this viral protein is capable of binding to the promoter regions and inducing transcription of some antisense transcripts independently of viral infection (Fig. 4). It should be mentioned, however, that in all assays (RNA-sequencing, RT-qPCR, Nanostring), the levels of antisense transcripts were considerably higher upon infection compared to ICP4 overexpression. While this could be due to technical issues, such as the activity and amount of the ectopically expressed ICP4, it might also indicate that other factors enhance the activity of ICP4 during infection. On a side note, no evidence for the involvment of viral ICP27 was found in recently published [49] RNA-sequencing data following overexpression of this protein (data not shown).

Taken together, the results indicate that different classes of antisense transcripts are induced by different mechanisms. While some of the antisense transcripts appear to be exclusive for the *Herpes simplex* genus, others may be more broadly conserved as they are observed in VZV- and partially in CMV-infected cells.

One of the most highly expressed antisense transcripts was BBC3as, which overlaps about half of the BBC3 gene body. Its TSS lies in the last intron of the BBC3 sense transcript. FISH showed strong nuclear foci comparable to the abundant lncRNA NEAT1, with a size and intensity that represents accumulation of multiple molecules. In addition, we found three such foci in infected HeLa cells, which are triploid at this locus [41], and two foci in most infected NHDF cells (Fig. 5b). Both observations are compatible with BBC3as accumulating at its site of transcription, maybe by binding to nascent BBC3 sense mRNA. To test whether BBC3as transcriptionally antagonizes transcription in the sense direction, we first analyzed gene expression in HSV-1-infected cells treated with the virus replication inhibitor PAA. Upon PAA treatment, infection progresses much more slowly, and the host cell shutoff was attenuated. In such conditions, we observed that the BBC3 mRNA levels decreased quickly, and that the BBC3as was transcribed at greater levels (Fig. 3b). The analysis of the 4sU-seq time course data, which showed how the syntheses of BBC3 sense and antisense transcripts are anti-correlated (Fig. 5c), confirmed this observation. Interestingly, we also observed a slight induction of the BBC3 sense RNA immediately upon infection, as in the RT-qPCR and Nanostring experiments (Figs. 2b and 3c; Additional file 1: Figure S2a). This is not unexpected, as the infection initially triggers apoptosis, which is then actively counteracted by the virus [38]. Furthermore, we provide evidence that the BBC3as downregulates transcription of the BBC3 mRNA on the transcriptional level (Fig. 5g–i). Taken together, this could indicate a completely novel and unexpected mechanism of how HSV-1 impairs apoptosis: by inducing an antisense transcript that transcriptionally downregulates the potent pro-apoptotic BBC3. A similar mechanism was recently shown in the plant *Arabidopsis thaliana*, where the COOLAIR antisense transcript blocks transcription of the corresponding sense transcript FLC *in cis* [50]. Notably, the BBC3 gene experiences transcriptional regulation by CTCF, which binds to the first half of the BBC3 gene, thereby inhibiting mRNA processing [45]. The excised fragment in the last intron in the BBC3as knockout cells appears, however, to be outside this region, also judged by the occurrence of CTCF binding sites [51]. This result indicates that the CTCF regulation is not affected in the cell lines used in our study.

Many of the antisense transcripts observed here start in close proximity to the transcription start sites of their respective host genes, giving rise to divergent or convergent transcripts. The cause, importance and function of these so-called bi-directional promoters is currently under discussion [11, 52]. In the course of this study, we asked two questions in this context: to what extent are the antisense transcripts transcribed in uninfected cells? And can the antisense transcripts induced by HSV-1 infection also be found in other biological settings? For an initial investigation of the first question, we analyzed published NET-seq data, nucleoplasmic RNA upon exosome depletion, and histone marks. Although the antisense transcripts are not detected in steady-state or newly synthesized RNA (4sU-seq), 75% of them are strongly transcribed in uninfected HeLa cells according to the NET-seq data (Fig. 2d). Furthermore, upon exosome depletion, e.g., BBC3as becomes also visible in nucleoplasmic RNA. On a similar note, we compared histone marks between induced and non-induced divergent and convergent antisense transcripts and found that the profiles look essentially the same (Fig. 4).

This could indicate that, also in human cells, antisense transcripts are synthesized, and their expression can vary between conditions, as also shown for the antisense transcripts belonging to the NF-kappaB transcription factor family. For example, BBC3as, which might counteract transcription of the BBC3 mRNA, could be induced upon HSV-1 infection or in cancerous cell lines such as HeLa to prevent apoptosis. Still, the question remains why the antisense transcripts becomes detectable in steady-state poly(A) + RNA only upon infection. Investigating this in the context of the virus infection could provide highly interesting insights in RNA processing and degradation of antisense RNA and gene regulation by lncRNAs.

## Conclusions

Our results indicate that HSV-1 infections provide a highly valuable and broadly and easily applicable tool to induce a wide range of antisense transcripts. Our study therefore provides a basis for further investigations of this type of transcript in other biological settings.

## Methods

### Cell culture and viruses (HeLa, WI-38, and NHDF cells)

HeLa cells and HeLa BBC3 as promoter knockout cells were maintained in Dulbecco’s modified Eagle medium (DMEM) supplemented with 10% fetal bovine serum (FBS), 100 units/ml penicillin, and 100 μg/ml streptomycin at 37 °C and 5% CO_2_. Normal human dermal fibroblasts (NHDFs) and WI-38 fibroblasts were cultured in minimal essential medium (MEM) supplemented with 10% FBS, 100 units/ml penicillin, and 100 μg/ml streptomycin. Wild-type HSV-1 was derived from a patient isolate and was obtained from Department of Virology, Saarland University Medical School, Homburg, Germany. The virus was propagated in HeLa cells or NHDF cells. At 72 hpi, viral supernatant was sterile-filtered through a 0.45 μm pore size filter and stored at −80 °C. Viral titers were determined by plaque assay. Briefly, HeLa cells were inoculated with 500 μl of virus-containing supernatant and serial dilutions in DMEM and incubated for 60 minutes at 37 °C and 5% CO_2_. The supernatant was then replaced by fresh complete DMEM containing 2% methylcellulose. Four to five days post-infection cells were fixed with 4% paraformaldehyde and stained with crystal violet. The PFU was then determined by counting the visible plaques and the experiments were carried out in triplicate. Cells were infected with an MOI of 10.

### Plasmids and transfections

Plasmid pEYFP-ICP4 was a kind gift from Chris Boutell [53]. For overexpression of the BBC3as transcript, a genomic region spanning the first 2191 bases of the antisense transcript was amplified using primers BBC3as_pcdna_fwd1/BBC3as_pcdna_rev2 and inserted into pcDNA3.0. Transfections were performed using FuGene HD (Promega). The plasmid expressing the ICP4-3xflag construct was derived from pEYFP-ICP4 by first replacing the EYFP by the oligos EYFP_NheI_SalI_fwd and EYFP_NheI_SalI_rev after NheI/SalI digestion, and then insertion of the ICP4Cterm-3xflag fragment using SgsI/KpnI (Additional file 3: Table S2).

### RNA isolation and sequencing

RNA was extracted from cultured cells with Trizol (Thermo Fisher Scientific) and purified from the aqueous phase using the RNA Clean & Concentrator 25 kit (Zymo Research). For sequencing from WI-38 cells, poly(A) RNA was selected using the Dynabeads mRNA DIRECT Kit (Thermo Fisher Scientific). Sequencing libraries were prepared using the NEXTflex Directional RNA-Seq Kit dUTP-based (Bioo Scientific) and sequenced on a Illumina HiSeq 2000 device. After trimming the adapter sequence AGATCGGAAGAGCACACGT, about 25–30 million mappable reads per sample were obtained. For sequencing from EYFP or EYFP-ICP4 transfected HeLa cells as well as HSV-1-infected HeLa and BBC3as knockout cells, poly(A) RNA sequencing libraries were prepared using the TruSeq Stranded mRNA Library Prep Kit (Illumina). For the HeLa infection time-course samples, a 5% *D. melanogaster* spike-in was added on the level of total RNA. EYFP/EYFP-ICP4 samples were sequenced on an Illumina NextSeq 500 device; infected HeLa samples were sequenced on a Illumina HiSeq 4000 device. After trimming the adapter sequence GATCGGAAGAGCACACGT, about 21–25 million mappable reads per sample were obtained for the EYFP/EYFP-ICP4 samples, and 12–18 million mappable reads for the HeLa/BBC3as knockout samples.

For visualization of RNA-sequencing data, alignment files were normalized by the number of mapped reads, and, after conversion to BigWig files, displayed using IGV [54]. Coverage in the minus direction is displayed as negative values. To compare different samples, the scale was set to the same values for the same strand within a group of samples. Such groups are, e.g., human foreskin fibroblast (HFF) cells on one side and WI-38 cells on the other side in Fig. 1, or WI-38 cells on one side and the HeLa wild-type/BBC3as knockout cells on the other side in Fig. 5.

### Bioinformatical identification and definition of antisense transcripts

Data for definition of antisense transcripts were acquired from the herpes-infected 4sU time-course data [8]. For transcript definition, we used all samples except 6–7 and 7–8 hpi. The data were mapped onto the hg19 reference using TopHat2 [55]. Because of the unique transcriptomic profiles in human cells infected with herpes simplex virus, antisense transcripts were defined on the coverage tracks of the HSV-1-infected 4sU samples using a custom running sum algorithm, followed by extensive filtering. For this purpose, all regions that overlapped known Ensembl protein coding, pseudogene, miRNA, lincRNA, and snoRNA loci were first set to have coverage of 0. Regions overlapping lincRNAs which have a TSS within 2 kb upstream of known protein coding genes were regarded as wrongly annotated antisense genes and were not filtered out. To locate the expressed loci, a running sum algorithm was run on the aforementioned tracks (in a strand-specific fashion). The algorithm scans the genome base by base. When it encounters a base that has coverage > 0, it marks a start of the putative region and increments a counter by a predefined value. With each base with coverage > 0 the counter is incremented, and with each base with coverage = 0 the counter is decremented by a predefined value. When the counter reaches 0, it marks the end of a putative region. The transcript definition was done for each timepoint individually. All transcripts that are supported by at least one read per base were kept for further analysis. The regions resulting from individual stages were merged in the following way. 1) The start of the region was set as the TSS that was supported in most of the time-course samples. 2) The width of the region was defined as the median widths of all the putative transcripts that overlapped a certain locus.

Putative antisense regions were filtered in the following way: all regions that were transcribed 1 kb downstream from the transcription termination sites of Ensembl annotated genes were marked as read-through transcripts and were omitted from further analysis.

All regions with length < 100 bp were omitted from further analysis.

### Annotation and expression analysis

Antisense transcripts were classified into three classes based on the location of the TSS in respect to the sense transcript:

- Divergent—TSS of the antisense transcript is located within 10 kb upstream of the sense gene
- Convergent—TSS of the antisense transcript is located 10 kb downstream of the sense gene
- Internal—TSS of the antisense transcript is located in the gene body of the antisense transcript, more than 10 kb downstream from the gene TSS.

### Annotation with CAGE data and lincRNA base

TSSs of antisense transcripts were overlapped with Fantom5 CAGE data to ascertain how many regions are known to produce capped transcripts. Additionally, the transcripts were overlapped with known lincRNAs from lincRNAdb.

### Analysis of antisense expression

To estimate the expression of the antisense transcripts, we first filtered the mapped reads to contain only properly paired read pairs. We then counted the number of reads that overlap each of the antisense transcripts from each of the 4sU and Ribo Zero RNA-seq samples from Rutkowski et al. [8]. The final expression estimates were obtained by normalizing counts for transcript length, and library size, by calculating the size factors from all protein coding genes. The expression for each timepoint was obtained by taking the log2 of timepoint expression/uninfected sample.

### RT-qPCR analysis

For RT-qPCR from total RNA, DNase digestion before the RT was applied to minimize background using DNase I, Amplification Grade (Thermo Fisher Scientific). Per reaction, 1 μg total RNA was used and transcribed using SuperScript III with random hexamer primers (both Thermo Fisher Scientific) according to the manufacturer’s protocol. For the fractioned, 4sU labeled samples, DNase treatment was not applied and 80 ng RNA was used per RT reaction. For qPCR, Power SYBR Green PCR Master Mix (Thermo Fisher Scientific) was used together with 250 nM of each primer and 1:40 final dilution of the RT reaction. Primers used for qPCRs are detailed in Additional file 3: Table S2. Minus RT values and standard curves are shown in Additional file 4: Table S3.

For all experiments, four measurements were done for two biological replicates. Data were normalized to the indicated timepoint/sample using the ∆∆Ct method [56].

### HFF cells, PAA, and knockout viruses

HFF cells were cultured and infection with wild-type virus was performed as described previously [8]. Cells were incubated with wild-type HSV-1 and FXE ICP0 knockout virus for 15’ and n12 ICP4 knockout virus (ICP4KO) for 60’. Where applicable, 300 μg/ml PAA (Sigma-Aldrich, catalog number 284270) was added in conditioned medium when the inoculum was removed. The n12 ICP4 knockout virus [57] was a kind gift from Neal DeLuca, and the FXE ICP0 knockout [58] virus was a kind gift from Stacey Efstathiou.

### Digital gene expression assays/Nanostring

Gene expression counts in virally infected cells were determined using nCounter Elements TagSets (NanoString) and an nCounter SPRINT profiler. Probe A and B sequences were custom designed with support from NanoString; oligonucleotides were obtained from IDT (Additional file 5: Table S4). Hybridizations were carried out for 18 h. Raw data were exported directly from the SPRINT profiler and normalized using the slope of the linear regression for the positive control values as normalization factor. All normalized values are shown in Additional file 6: Table S5.

### Chromatin immunoprecipitation

Chromatin immunoprecipitation (ChIP) was performed as previously described [59, 60] with the following modifications. Per sample, 40 μg of crosslinked DNA (as determined after reverse crosslinking, protease treatment, phenol extraction, and ethanol precipitation) was used as input. ICP4-3xflag crosslinked to chromatin was precipitated by incubation of sheared chromatin with anti-flag M1 agarose affinity gel (Sigma-Aldrich, catalog number A2220) for 3 h at 4 °C. Yields in the eluates were about 3 ng DNA for control samples (untransfected cells) and 10 ng for ICP4-ChIP samples, as determined by measurements with the Qubit DNA HS assay (Thermo Fisher, catalog number Q32851). For qPCR, 10 ng of DNA template for input and 50 pg for eluate samples was used, as described in the “RT-qPCR analysis” section above.

### Single-molecule RNA fluorescence in situ hybridization

Oligonucleotide probes were designed using the Stellaris Probe Designer (https://www.biosearchtech.com/support/tools/design-software/stellaris-probe-designer). Customs probes listed in Additional file 7: Table S6 were ordered either coupled to Quasar 570 (Cy3 replacement) or Quasar 670 (Cy5 replacement) from LGC Biosearch technologies, as well as probes for NEAT1 (catalog number SMF-2036-1). Cells were seeded in 12-well format on gelatin-coated coverslips; 24 h later cells were infected with HSV-1 and fixed with 4% paraformaldehyde at 0, 4, and 6 hpi. Cells were washed once with PBS containing Ca and Mg and fixed in 4% EM-grade formaldehyde in PBS containing 1 mM Ca and Mg for 10 minutes at room temperature, transferred to ice, washed once with PBS, and stored up to several weeks in 70% ethanol at 4 °C.

Hybridizations were performed as described [61], with minor modifications. Stellaris probes were hybridized overnight at 37 °C in 2 × SSC, 10% dextran sulfate, 0.4% SDS with a stringency of 10% formamide at a concentration of 12.5 to 50 nM. After hybridization, unbound probes were washed away two times, and 5 ng per ml DAPI nuclear stain was included in the second wash. Images were acquired on an inverted Nikon Ti microscope with an Andor iXon Ultra DU-888 camera in CCD mode (1 MHz, conversion gain 2), a 60x NA 1.4 oil objective and Nikon NIS-Elements Ar software (version X), using exposure times of 50 ms for DAPI and 300 to 500 ms in the other fluorescence channels at 50 to 100% intensity of the light source (Sola SEII). Groups of cells for imaging were chosen in the DAPI channel; Z-stacks with 0.3 μm spacing were acquired in all channels including EGFP (to control for non-specific signals generated by autofluorescence). Images were processed using open source software Fiji and merged by maximum intensity projection, as indicated in the figure legends.

### Promoter reporter assay

We used a modified pMIR-RNL TK vector (a dual firefly and renilla luciferase vector) described previously [62]. The CMV promoter in this plasmid was replaced by the predicted BBC3 antisense transcript promoter. The sequences inserted into this novel construct pMIR-AS are shown in Additional file 1: Figure S5. The predicted promoter sequence (~700 bp) and shorter sequences (~500 and 300 bp) were cloned in the newly modified pMIR-RNLTK. Typically, HeLa cells were seeded in 24-well format and transfected with 0.2 μg per well reporter vector using PolyFect (Qiagen) and assays were conducted in duplicate. After 48 h cells were infected with HSV-1 or mock infected (0 h) with DMEM. Cells were harvested at 0, 6, and 8 hpi. The ratio of firefly (reporter)/renilla (control) luciferase for each sample was determined (%RLU).

### Generation of BBC3as knockout cells

Optimal guide RNA sequences were determined using the CRISPR design website (crispr.mit.edu). The following gRNA sequences were used: TGCGCGGCTGGGTCGCTCCG (gRNA 5), GCTTTTACTCCGTATTTACC (gRNA 6), and TAGAAACACAAGAGCGCACC (gRNA 9). Templates for T7 transcription of candidate RNAs were prepared using oligos BBC3asprom5, BBC3asprom6, BBC3asprom9 as well as T7_tracrRNA (Additional file 3: Table S2) and the Phusion PCR polymerase (Thermo Fisher Scientific) to generate double-stranded DNA. In vitro transcription was performed using T7 polymerase (New England Biolabs, catalog number E2050S). For the in vitro cleavage assay using Cas9 (New England Biolabs, catalog number M0641S), DNA templates were amplified from HeLa genomic DNA using the following primers: BBC3asivtempl12345fw and BBC3asivtempl12345re for gRNA 5, BBC3asivtempl678fwd and BBC3asivtempl678rev for gRNA 6, and ivtemplC_fwd_3 and ivtemplC_rev_3 for gRNA 9. For expression of the gRNAs, we used plasmids pSpCas9(BB)-2A-GFP (pX458, Addgene plasmid 48138), a gift from Feng Zhang [63], and pU6-(BbsI)_CBh-Cas9-T2A-mCherry (px330-mCherry, Addgene plasmid 64324), a gift from Ralf Kuehn [64]. To prepare gRNA/Cas9 expression plasmids, oligos BBC3aspromg5inpxo1/BBC3aspromg5inpxo2 were inserted into BbsI-digested px330-mCherry and BBC3aspromg6inpxo1/BBC3aspromg6inpxo2 or BBC3aspromg9inpxo1/BBC3aspromg9inpxo2, respectively, into BbsI-digested px458.

Plasmids containing gRNA 5 and gRNA 9 or gRNA 6 and gRNA 9, respectively, were co-transfected into HeLa cells and double transfectants selected and single cells sorted into 96-well plates using FACS. Cells were recovered for 2 to 3 weeks and, after replication of plates, washed with PBS, detached with PBS/1 mM EDTA, and lysed with 0.5% Triton X-100 and 2 mg/ml Proteinase K in 30 mM Tris pH 8. The mixture was incubated for 10 minutes at 70 °C and the Proteinase inactivated by incubation at 95 °C for 10 minutes. The lysate was then used for qPCR using primers BBC3asCRIS_wtonly_fwd2 and BBC3asCRIS_wtonly_rev2, which detect only the wild-type allele, and either BBC3asCRIS_56only_fwd1/BBC3asCRIS_56only_rev1 or BBC3asCRIS_59only_fwd1/BBC3asCRIS_59only_rev1, which detect only mutant alleles. Ploidy of selected clones was then measured using the same primer pairs with BBC3as-2_fwd/BBC3as-2_rev for normalization from purified genomic DNA. For the experiments here, the homozygous knockout clones 56-D2 and 59-B3 were used.

### RNA fractionation

For transcription activity assays, nascent RNA was labeled for 20 minutes with 500 μM 4sU. After MTS-mediated biotinylation [65] from 80 μg of total RNA, labeled RNA was recovered as previously described [66].

### Analysis of histone modification data

Histone modification profiles were plotted in a region around the promoter of antisense and control genes in the following way. Genes that had a transcription start site within 5 kb of an antisense (novel or annotated) transcript or had expression < 10 RPKM in all time points of the 4sU data were removed from the analysis. From the resulting set, a random set of 1000 genes was chosen.

Antisense transcripts were marked as induced if they had a minimal fold change of 2 in at least one time point of the 4sU data.

Histone modification ChIP-seq signals were extracted in a region of ± 4 kb around the TSS of both the antisense transcripts and selected control genes. The signal for each TSS was scaled to have zero mean and a standard deviation of one, and averaged separately for each of the following groups: divergent antisense transcripts, convergent antisense transcripts, internal antisense transcripts, control genes.

## Additional files

Additional file 1: Figures S1–S5. ** Figure S1** Coverage profiles of antisense transcripts. **Figure S2** Validation of antisense transcripts. **Figure S3** Antisense transcripts with PAA, knockout virus, and HSV-2 infection [67]. **Figure S4** Antisense transcript promoters are already poised for transcription. **Figure S5** Ectopically expressed BBC3as localizes outside the nucleus. (PDF 4275 kb)

Additional file 2: Table S1. Antisense transcripts. (XLSX 1601 kb)

Additional file 3: Table S2. Oligos. (XLSX 43 kb)

Additional file 4: Table S3. qPCR additional data: minus RT values, standard curves. (XLSX 42 kb)

Additional file 5: Table S4. Nanostring probes. (XLSX 34 kb)

Additional file 6: Table S5. Nanostring counts. (XLSX 50 kb)

Additional file 7: Table S6. FISH probes. (XLSX 50 kb)
