# Supplementary material for: Widespread activation of antisense transcription of the host genome during herpes simplex virus 1 infection
Source: Genome Biol. 2017 Oct 31;18:209. doi: 10.1186/s13059-017-1329-5 (PMC5663069; doi:10.1186/s13059-017-1329-5)
Supplement: Supplementary file 1 — Figure S1 Coverage profiles of antisense transcripts. Figure S2 Validation of antisense transcripts. Figure S3 Antisense transcripts with PAA, knockout virus, and HSV-2 infection [67]. Figure S4 Antisense transcript promoters are already poised for transcription. Figure S5 Ectopically expressed BBC3as localizes outside the nucleus. (PDF 4275 kb) [file 13059_2017_1329_MOESM1_ESM.pdf]

Supplemental Figure 1, continued

A, continued

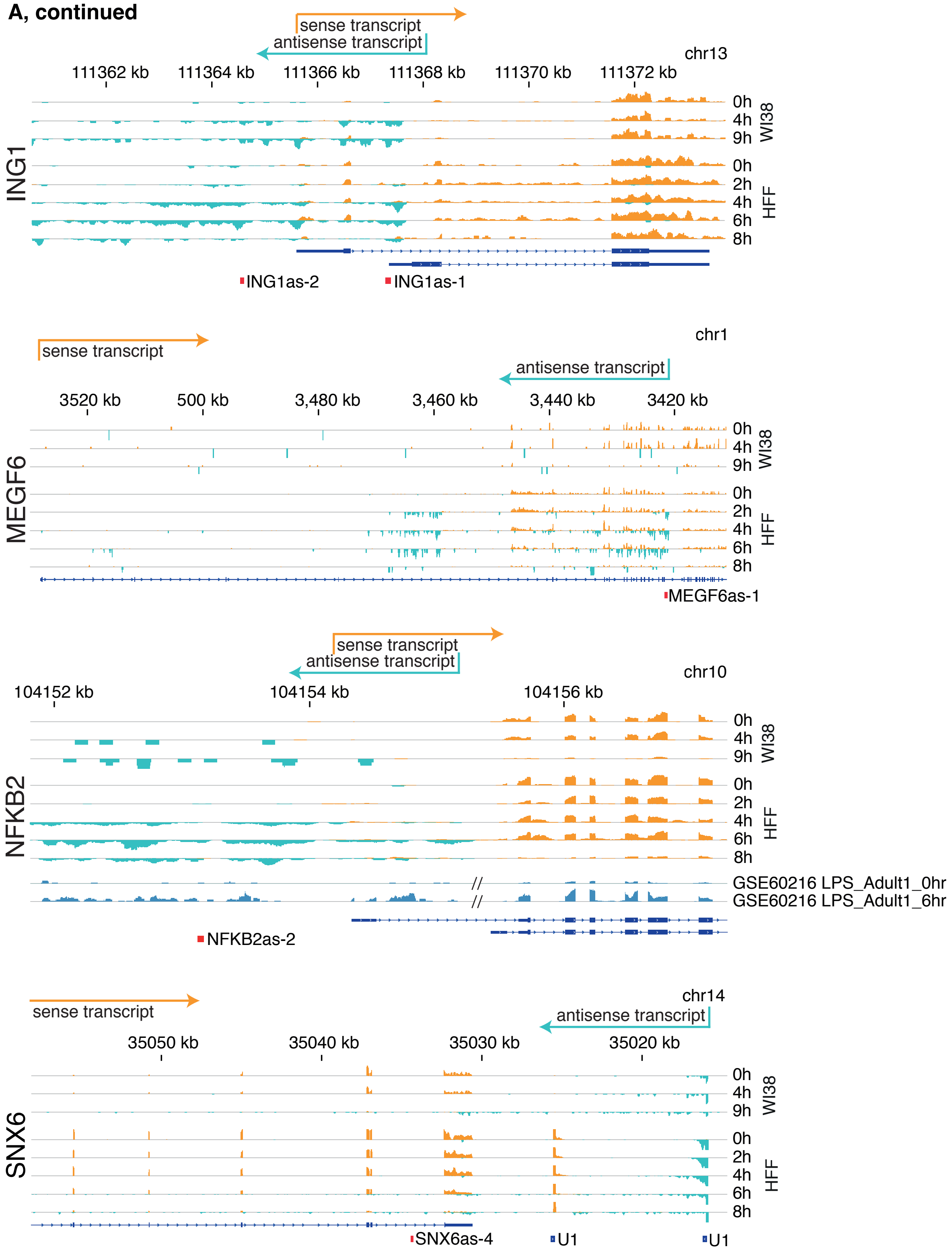

B

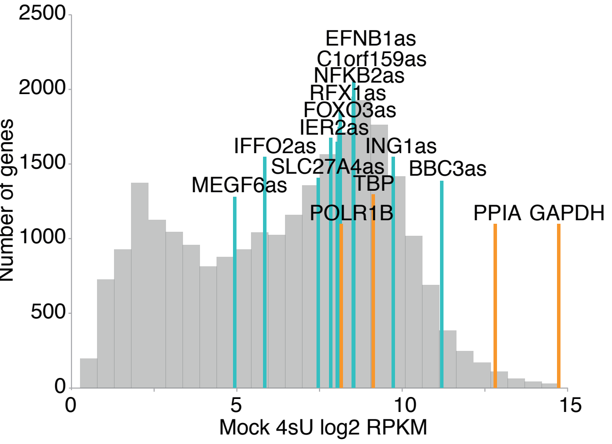

Supplemental Figure 1

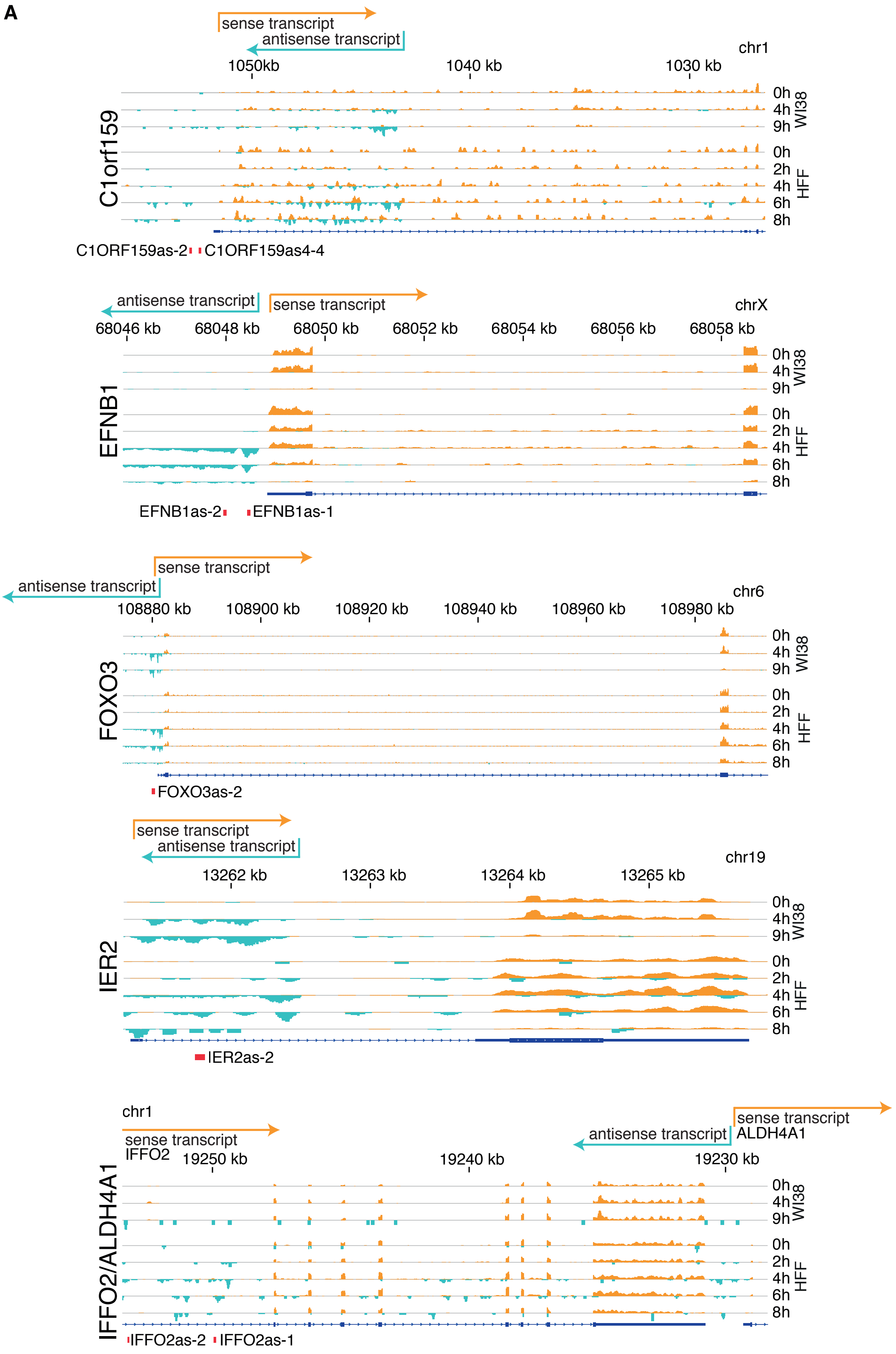

Figure S1 I Coverage profiles of antisense transcripts.
A: Examples of antisense transcripts. Coverage profiles for polyA selected (WI38 cells) and Ribozero treated (HFF cells) total RNA-sequencing data is shown. Sense genes are depicted in orange running left to right, antisense transcripts in petrol running right to left. Respective transcription starts and chromosome regions are indicated. Refseq annotations are shown in dark blue, location of RT-qPCR primer pairs in red.
B: Histogram of expression values. Log2 RPKM for all genes in the pulse-labeled mock sample are plotted as a histogram in grey, with selected housekeepings in orange. Maximal values for the antisense transcripts from the timecourse are marked in petrol.

Figure S2

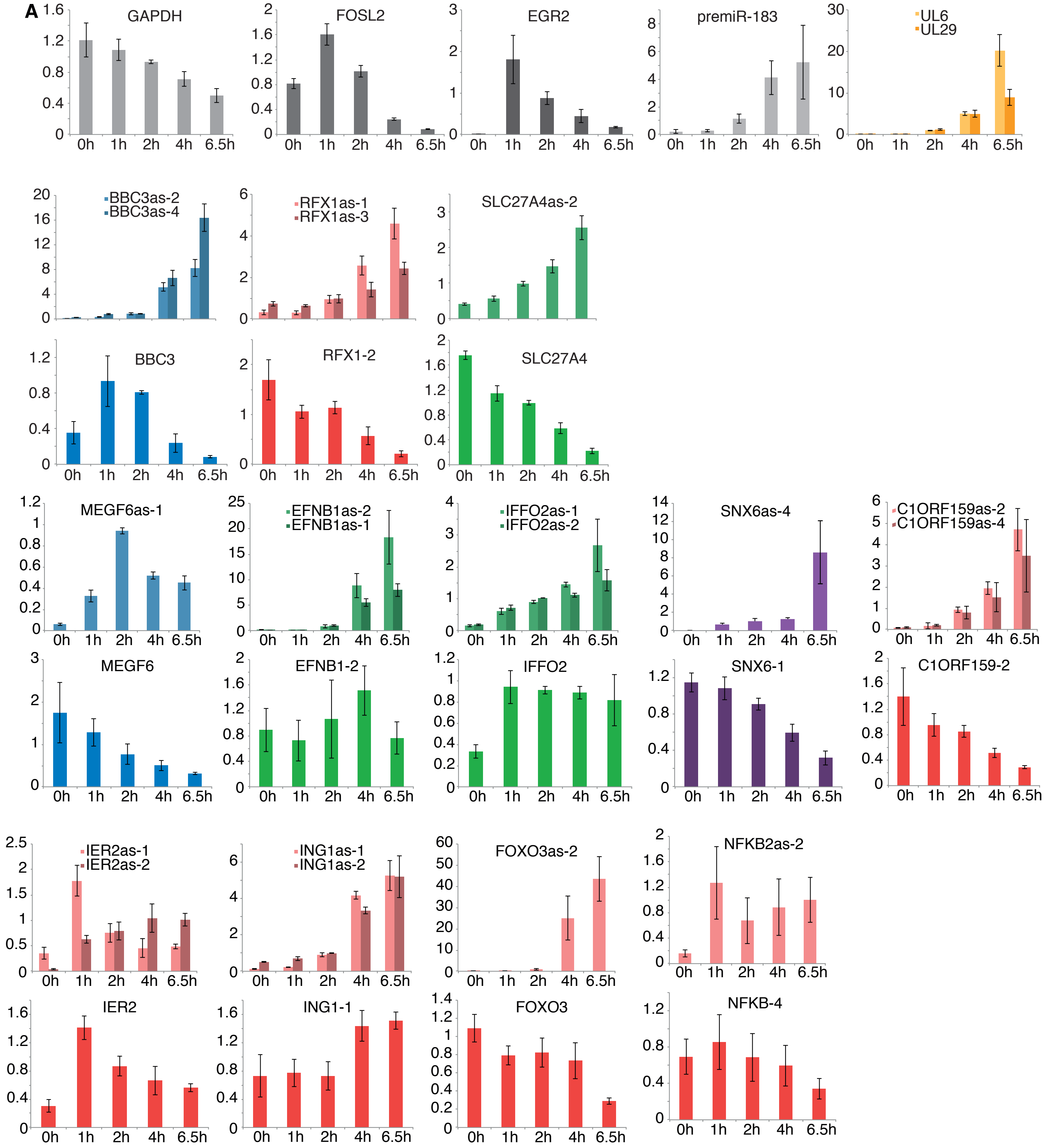

Figure S2 I Validation of antisense transcripts.

A: RT-qPCR, antisense transcripts and corresponding sense mRNAs. RNA was isolated from HeLa cells at different timepoints post infection and subjected to random hexamer directed RT followed by qPCR with primer pairs as indicated. Experiments were performed with two measurements each from two biological replicates and normalized to the 2hpi timepoint using the D. melanogaster spike-in RNA. Control bars represent standard deviations.

B: Nanostring nCounter assays, antisense transcripts and corresponding sense mRNAs. RNA was isolated from the three indicated cell lines at different timepoints post infection and used for Nanostring nCounter assays. Experiments were performed with one measurement each from two biological replicates and scaled to the 2hpi timepoint after normalization using the provided control spike-in. Control bars represent standard deviations.

C: Expression profiles of antisense and readthrough transcripts in comparison. Shown are median values (solid lines) and 25%-75% quantiles (shaded areas).

D: RT-qPCR on readthrough transcripts. Experiments were performed as in A, here measuring a SRSF2-JMJD6 fusion transcript (JMJD6 is about 8 kB downstream of SRSF2, in the same orientation), and a fusion between the second-last exon of SRSF3 and a newly emerging cryptic exon about 35kB downstream of SRSF3.

E: BBC3as is exosome sensitive. Coverage profiles for polyA RNA (HSV-1 infected WI38 cells) nucleoplasmic RNA (EXOSC3 depleted HeLa cells) RNA-sequencing data is shown. Sense genes are depicted in orange running left to right, antisense transcripts in petrol running right to left. Respective transcription starts and chromosome regions are indicated. Refseq annotations are shown in dark blue.

Figure S2, continued

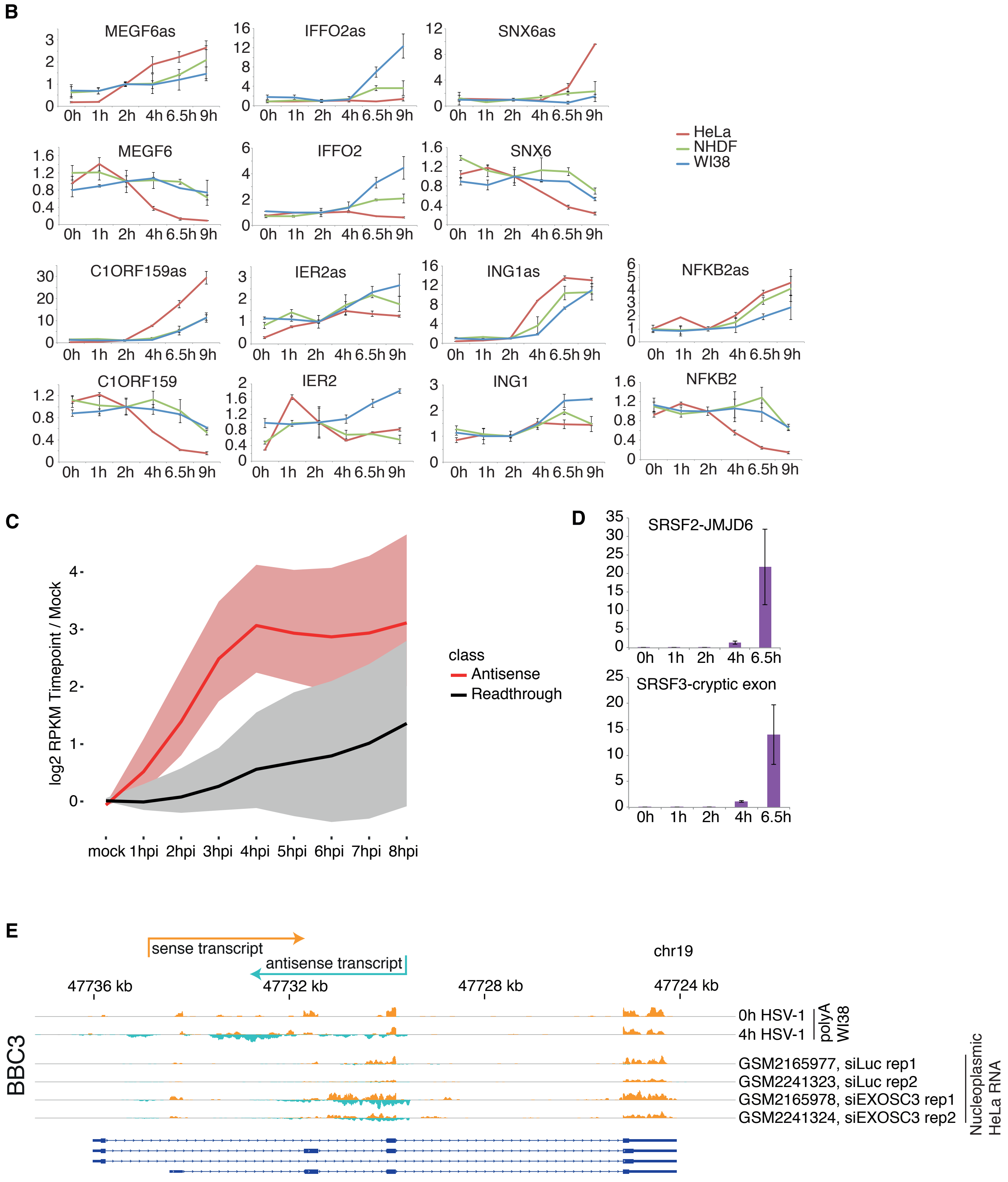

Figure S3

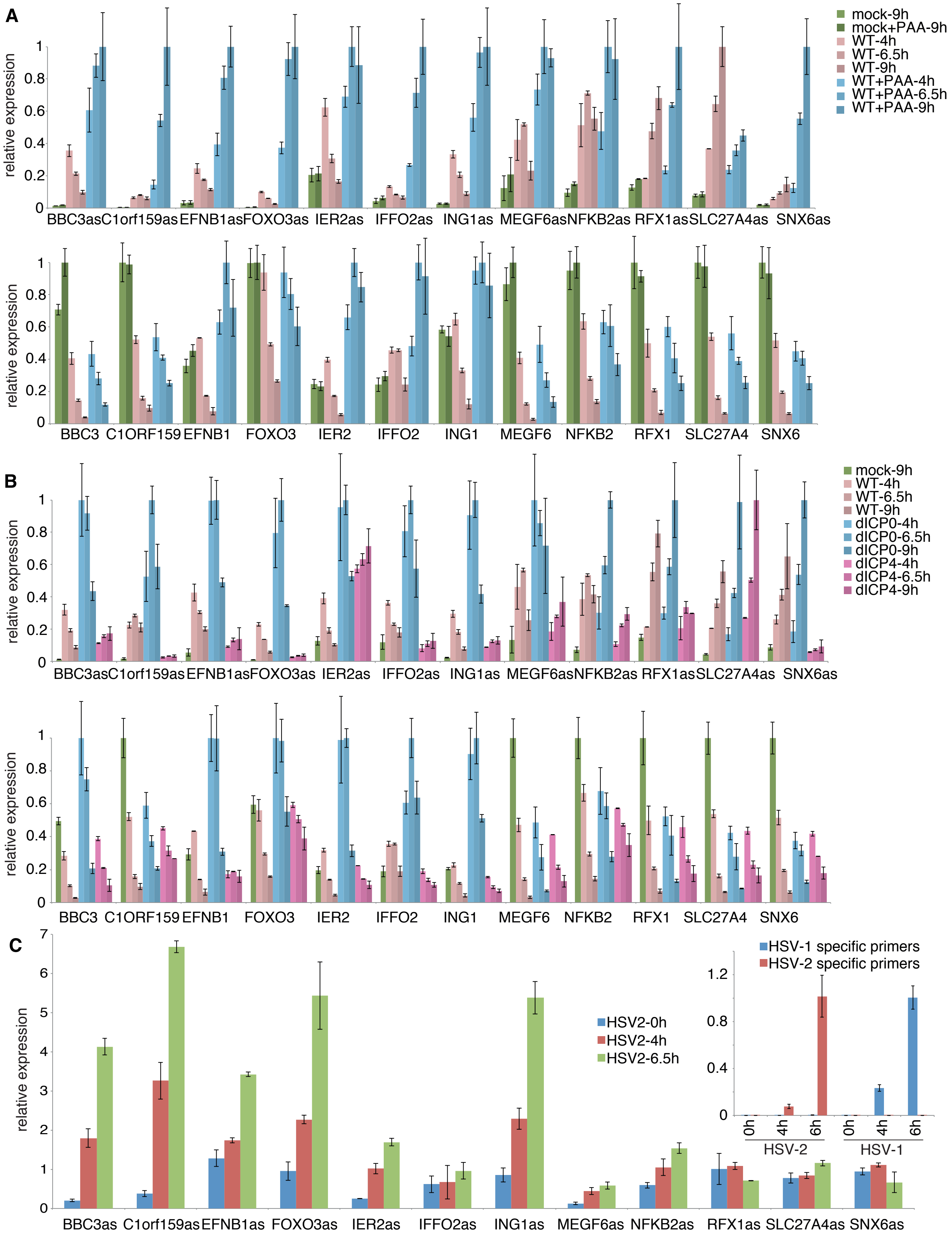

Figure S3 | Antisense transcripts with PAA, knockout virus, and HSV-2 infection

A: Nanostring nCounter profiling with the replication inhibitor PAA. RNA was collected from HFF cells at different timepoints post infection with our without the HSV-1 replication inhibitor PAA. Experiments were performed with one measurement each from two biological replicates and scaled to the 2hpi timepoint after normalization using the provided control spike-in. Control bars represent standard deviations. All values are scaled to the largest value for the same transcripts.

B: Nanostring nCounter profiling using ICP0 and ICP4 knockout viruses, as in A.

C: Nanonstring nCounter profiling of antisense transcripts in HSV-2 infected cells. RNA was collected from HeLa cells at different timepoints post infection. Experiments were performed with one measurement each from two biological replicates and scaled to the 2hpi timepoint after normalization using the provided control spike-in. Values are scaled to the 2h timepoint from HSV-1 infected HeLa cells. Insert: Identity of viruses was verified using published qPCR primers (Adelson et al. 2005)

Figure S4

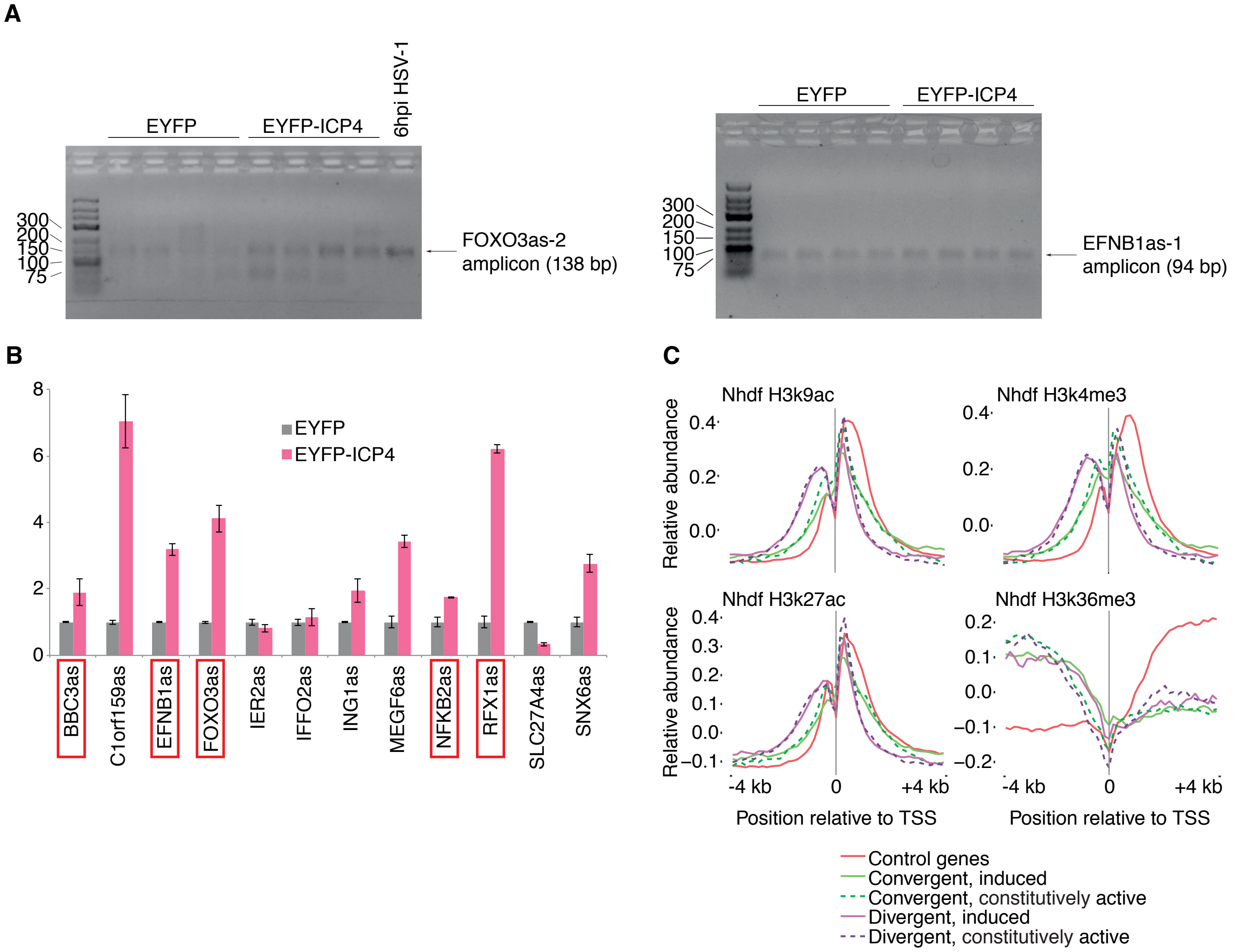

Figure S4 I Viral ICP4 induces antisense transcription from promoters that are already poised for transcription.
A: RT-qPCR amplicons. Agarose gels of the RT-qPCR products shown in Figure 4.
B: Nanostring analysis of antisense transcripts upon ICP4 overexpression. HeLa cells were transfected with either the EYFP control plasmid or EYFP-ICP4, and isolated RNA subject to Nanostrin nCounter analysis. Antisense transcripts which also showed induction in the RNA-seq data are indicated with red boxes.
C: Histone marks metaplots. Metaplots were generated for four histone marks around the transcription start sites of inducible and constitutively transcribed antisense transcripts, together with 1000 unidirectional control genes.

Figure S5

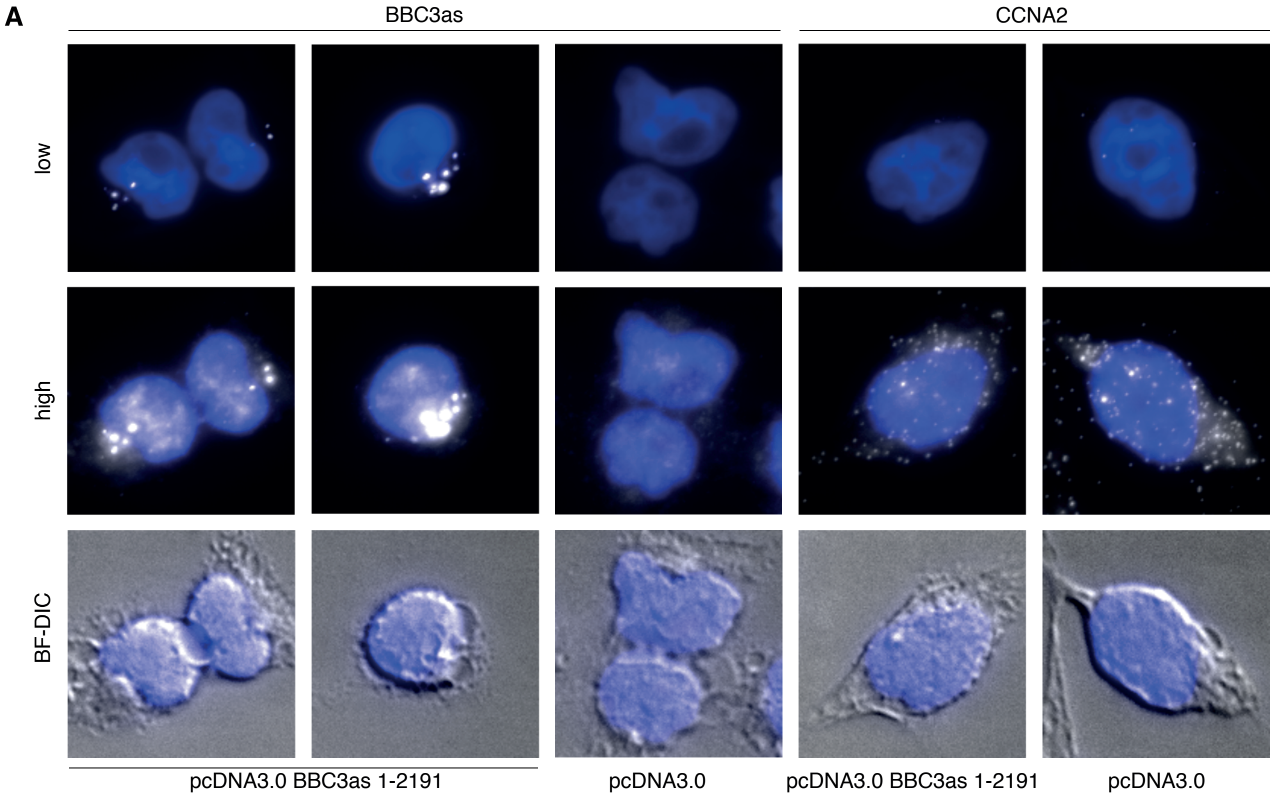

Figure S5 I Ectopically expressed BBC3as localizes outside the nucleus
A: Single-molecule FISH of BBC3as. The first 2191 basepairs of the BBC3as transcript were expressed from pcDNA3.0 and visualized using single-molecule FISH.
